# Genomic profiling of advanced cervical cancer to predict response to programmed death-1 inhibitor combination therapy: a secondary analysis of the CLAP trial

**DOI:** 10.1136/jitc-2020-002223

**Published:** 2021-05-17

**Authors:** Xin Huang, Minjun He, Hongyu Peng, Chongjie Tong, Zhimin Liu, Xiaolong Zhang, Yang Shao, Dongqin Zhu, Junli Zhang, Jiani C Yin, Fan Yang, Chunyan Lan

**Affiliations:** 1 Gynecologic Oncololgy, Sun Yat-sen University Cancer Center, Guangzhou, Guangdong, China; 2 Key State Laboratory of Oncology in South China, Sun Yat-sen University, Guangzhou, Guangdong, China; 3 Center for Bioinformatics, Sun Yat-sen University Cancer Center, Guangzhou, Guangdong, China; 4 Nanjing Geneseeq Technology Inc, Nanjing, China; 5 School of Public Health, Nanjing Medical University, Nanjing, Jiangsu, China

**Keywords:** immunotherapy, gene expression profiling, genetic markers, drug therapy, combination

## Abstract

**Background:**

The Camrelizumab Plus Apatinib in Patients with Advanced Cervical Cancer trial was a single-arm, phase II study that showed promising activity of the programmed death-1 (PD-1) inhibitor camrelizumab plus the vascular endothelial growth factor receptor-2 inhibitor apatinib in patients with advanced cervical cancer. However, the predictive biomarkers for treatment outcomes are unknown. In this study, we aimed to identify potential predictors of treatment response in PD-1 inhibitor combination therapy.

**Methods:**

Genomic profiling was performed on patients with available biopsy or surgical samples by targeted next-generation sequencing of 425 cancer-related genes in this preplanned, secondary analysis. Somatic alterations, including all non-synonymous mutations, and tumor mutational burden (TMB) were assessed for their predictive values in objective response rate, progression-free survival (PFS), and overall survival (OS).

**Results:**

A subset of 32 patients was included in this analysis. Top altered genes included *PIK3CA* (43.8%), *STK11* (25%), *FBXW7* (15.6%), and *PTEN* (15.6%). The PI3K/AKT pathway was among the most frequently dysregulated pathways, and its genetic alterations were identified in 68.8% of patients. *PIK3CA* (PFS HR 0.33, p=0.05; OS HR 0.23, p=0.04) and *PTEN* (PFS HR 3.71^e-09^, p=0.05; OS HR 3.64^e-09^, p=0.08) alterations were associated with improved outcomes. PI3K/AKT pathway genetic alterations showed improved predictive power compared with either *PIK3CA* or *PTEN* alterations alone (PFS HR 0.33, p=0.03; OS HR 0.25, p=0.02), while *ERBB3* mutations (PFS HR 34.9, p<0.001; OS HR 19.8, p<0.001) correlated with poor survival. TMB-high (≥5 mut/Mb) was associated with prolonged PFS (HR 0.26, p<0.01) and OS (HR 0.31, p=0.05). Multivariate analysis showed *ERBB3* mutations (PFS p=0.01, OS p<0.001), PD-L1 positive (PFS p=0.01, OS p=0.05), and high TMB (PFS p=0.01, OS p=0.05) remained significantly associated with survival.

**Conclusions:**

We uncovered that genetic alterations in *PIK3CA*, *PTEN*, *ERBB3*, and PI3K/AKT pathway, as well as TMB, could be novel predictive biomarkers in patients with cervical cancer treated with PD-1 inhibitor combination therapy.

**Trial registration number:**

NCT03816553.

## Background

Cervical cancer is the fourth most prevalent cancer and the fourth leading cause of cancer-related deaths in women worldwide.^1^ In China, 98,900 new cases of cervical cancer and 30,500 cervical cancer-related deaths were estimated in 2015.^2^

Platinum-based chemotherapy is the first-line treatment for patients with metastatic, recurrent, or persistent cervical cancer.^3^ The prognosis of patients who progressed after first-line therapy is poor. Recently, immune checkpoint inhibitor (ICI) therapy, including cytotoxic T-lymphocyte antigen 4, programmed death-1 (PD-1), and programmed death-ligand 1 (PD-L1) inhibitors, has revolutionized the treatment of several cancers.^4 5^ Pembrolizumab, a PD-1 inhibitor, has been approved as second-line treatment for patients with advanced PD-L1-positive cervical cancer. However, including those with cervical cancer, only a small subset of patients respond to ICI therapy.^6 7^ It is important to explore validated biomarkers that will help identify patients who are more likely to respond to ICIs.

PD-L1 is the first and most widely investigated biomarker for predicting response to ICIs.^8^ Despite its broad utility, PD-L1 expression alone is insufficient for predicting ICI response. The KEYNOTE-028 and KEYNOTE-158 trials showed that PD-L1 expression was predictive in only 17% and 14.6% of the patients with advanced cervical cancer treated with pembrolizumab, respectively.^6 7^ In the Checkmate-358 trial, 10 patients with PD-L1-positive advanced cervical cancer who received nivolumab monotherapy achieved a response rate of 20%.^9^ In addition to PD-L1, the tumor microsatellite instability (MSI) status and mismatch repair deficiency (dMMR) have been demonstrated to correlate with ICI therapy response across cancer types. Analysis of the MSI status across 39 cancer types showed microsatellite instability-high (MSI-H) in 2.6% of patients with cervical cancer.^10^ Tamura *et al* analyzed the MSI status of 20 patients with cervical cancer treated with nivolumab.^11^ Despite a response rate of 25%, none of the patients were classified with MSI-H. It should be noted that the MSI-H/dMMR status can be assessed by multiple methods, thereby increasing its variability.^12^ Thus, more accurate predictors and additional complementary biomarkers for ICI response in cervical cancer are warranted.

Next-generation sequencing (NGS)-based gene panel tests are commonly used in clinical practice to identify suitable patients for targeted therapy. It also provides valuable information, particularly on specific somatic alterations and tumor mutational burden (TMB), to predict response to ICI therapy. However, there have been limited studies on genomic profiling in cervical cancer. The predictive value of genomic alterations for ICI response in cervical cancer is unknown. Previously, we reported the outcomes of the Camrelizumab Plus Apatinib in Patients with Advanced Cervical Cancer (CLAP) trial on the efficacy and safety of camrelizumab (PD-1 inhibitor) combined with apatinib (vascular endothelial growth factor receptor-2 (VEGFR-2) inhibitor) in patients with advanced cervical cancer.^13^ The CLAP trial revealed that camrelizumab plus apatinib had promising antitumor effects in patients with advanced cervical cancer. In the current study, we conducted a preplanned, secondary analysis of genomic profiles in this cohort by targeted sequencing to identify potential predictive biomarkers of treatment response for this combination therapy.

## Methods

### Clap study design

The CLAP trial is a multicenter, single-arm, phase II study on patients with metastatic, recurrent, or persistent cervical cancer treated with camrelizumab plus apatinib. The design and results have been previously reported.^13^ The present study aimed to assess the genomic profiles of patients in the CLAP trial and identify genetic predictors of treatment response. Approval for this secondary analysis was obtained from the Research Ethics Board at each site, and all subjects signed informed consent forms.

### DNA extraction and targeted NGS

Formalin-fixed paraffin-embedded (FFPE) tumor samples obtained through biopsy or surgical excision were collected for targeted gene panel sequencing. Samples with ≥20% tumor cell content were qualified and included. Genomic DNA from FFPE sections and whole blood control samples were extracted with the QIAamp DNA FFPE Tissue kit and DNeasy Blood and Tissue Kit (Qiagen, USA), respectively. Sequencing libraries were prepared using the KAPA Hyper Prep Kit (KAPA Biosystems) following the manufacturer’s instructions for different sample types. Customized xGen lockdown probes (Integrated DNA Technologies) targeting 425 cancer-relevant genes (Geneseeq) were used for hybridization enrichment. The capture reaction was performed with Dynabeads M-270 (Life Technologies), xGen Lockdown hybridization, and wash kit (Integrated DNA Technologies) following the manufacturer’s protocols. Captured libraries were obtained using on-bead PCR amplified with Illumina p5 (5′ AAT GAT ACG GCG ACC GA 3′) and p7 primers (5′ CAA GCA GAA GAC GGC ATA CGA GAT 3′) in KAPA HiFi HotStart ReadyMix (KAPA Biosystems), followed by purification using Agencourt AMPure XP beads. Libraries were quantified with quantitative PCR using the KAPA Library Quantification kit (KAPA Biosystems), and the size was determined using Bioanalyzer 2100 (Agilent Technologies). The target-enriched library was then sequenced on the HiSeq4000 NGS platform (Illumina) following the manufacturer’s instructions. The mean coverage depth was 167X for the whole blood control samples and 1156X for tumor tissues.

### Sequence alignment and data processing

Base calling was performed on bcl2fastq V.2.16.0.10 (Illumina) to generate sequence reads in the FASTQ format (Illumina 1.8+encoding). Quality control was performed using the Trimmomatic software. High-quality reads were mapped to the human genome (hg19, GRCh37 Genome Reference Consortium Human Reference 37) using the Burrows-Wheeler Aligner (BWA) V.0.7.12 with Burrows-Wheeler Aligner's maximal exact matches (BWA-MEM) algorithm and default parameters to create SAM files. Picard V.1.119 was used to convert SAM files to compressed BAM files, which were then sorted according to chromosome coordinates. The Genome Analysis Toolkit (GATK, V.3.4–0) was used to locally realign the BAM files at intervals with insertions/deletion (indels) mismatches and recalibrate base quality scores of reads in BAM files.

### Germline/SNVs/indels/CNVs detections

Single-nucleotide variants (SNVs) and indels were identified using VarScan2, with a minimum variant allele frequency threshold set at 0.01 and p value threshold for calling variants set at 0.05 to generate Variant Call Format files. All SNVs/indels were annotated with ANNOVAR, and each SNV/indel was manually checked on the Integrative Genomics Viewer. SNVs and indels were further filtered with the following parameters: (1) minimum read depth=20; (2) minimum base quality=15, (3) minimum variant supporting reads=5, (4) variant supporting reads mapped to both strands, (5) strand bias no greater than 10%, (6) if present in >1% population frequency in the 1000 g or ExAC database and (7) through an internally collected list of recurrent sequencing errors using a normal pool of 100 samples. The sequencing assay has been validated in compliance with college of American pathologists (CAP) and clinical laboratory improvement amendments (CLIA) with a limit of detection of 1% VAF. Finally, the levels of 8-oxoguanine and deamination artifacts were estimated using GATK4 CollectSequencingArtifactMetrics and GATK4 FilterByOrientationBias was used to remove these artifacts. Germline mutations were filtered out by comparing them to the patient’s whole blood controls. Copy number variations (CNVs) were analyzed with CNVkit.^14^ Depth ratios of above 2.0 and below 0.6 were considered as CNV gain and CNV loss, respectively. All non-synonymous mutations, including missense, nonsense, indel, splicing, CNV and fusion, were analyzed. Altered genes were also grouped into their respective Kyoto Encyclopedia of Genes and Genomoes (KEGG) pathways. The genes that grouped into the PI3K/AKT pathway included *PIK3CA*, *PTEN*, *AKT*, *mTOR*, *RPTOR*, *TSC1*, *TSC2*, *LKB1*, *MAP2K1* and *MAP2K2* (online supplemental figure 3).

10.1136/jitc-2020-002223.supp1 Supplementary data

### Immunohistochemical analysis of PD-L1 expression

Immunohistochemical analysis of PD-L1 expression has been previously reported in the CLAP study. In brief, PD-L1 expression was assessed in archival tumor samples using the PD-L1 immunohistochemistry 22C3 pharmDx assay (Agilent Technologies, Santa Clara, California, USA). The PD-L1 protein expression was measured using combined positive score (CPS), defined as the number of PD-L1 staining cells (tumor cells, lymphocytes, and macrophages) divided by the total number of viable tumor cells, multiplied by 100. The specimen was considered to be PD-L1 positive if CPS is ≥1.

### Statistical analysis

For survival analyses, Kaplan-Meier curves were compared using the log-rank test, and the HRs with 95% CIs were calculated using the Cox proportional hazards model. The reverse Kaplan-Meier method was used to calculate the median follow-up time. Fisher’s exact test was used to compare proportions of groups, and the OR with 95% CI for response to treatment were provided. A two-sided p<0.05 was considered significant for all tests unless indicated otherwise. All statistical analyses were performed using R V.3.3.2.

## Results

### Patient characteristics

A subset of 32 patients was included in this analysis. These patients presented with similar baseline characteristics when compared with the whole CLAP population (table 1). Of the patients, 65.6% had squamous cell carcinoma (SCC) and the remaining had adenocarcinoma; 78.1% were PD-L1-positive, and 18.8% were PD-L1-negative.

**Table:** Table1. Baseline characteristics of patients included in the CLAP study and in the current analysis

Characteristics	CLAP study population *N* (%)	Subpopulation in the current analysis *N* (%)
Patients	*N=*45	*N*=32
Age (years)		
˂ 60	39 (86.7)	29 (90.6)
≥60	6 (13.3)	3 (9.4)
Median (range)	51 (33–67)	50 (33–63)
Median time from initial cancer diagnosis to study enrollment, months (range)	21.5 (3.7–92.1)	21.5 (3.7–92.1)
ECOG performance status		
0	10 (22.2)	7 (21.9)
1	35 (77.8)	25 (78.1)
Histology		
Squamous cell carcinoma	30 (66.7)	21 (65.6)
Adenocarcinoma	15 (33.3)	11 (34.4)
Target lesion size, mm		
Median (range)	41 (15–131)	41 (15–95)
Previous radiotherapy	40 (88.9)	28 (87.5)
Adjuvant radiotherapy	25 (55.6)	19 (59.4)
Curative radiotherapy	10 (22.2)	7 (21.9)
Palliative radiotherapy	5 (11.1)	2 (6.3)
Number of previous systemic therapies		
1	19 (42.2)	14 (43.8)
2	19 (42.2)	12 (37.5)
≥3	7 (15.5)	6 (18.7)
P16 expression		
Positive	29 (64.4)	21 (65.6)
Negative	10 (22.2)	10 (31.3)
Unknown	6 (13.3)	1 (3.1)
PD-L1 expression status		
Positive	30 (66.7)	25 (78.1)
Negative	10 (22.2)	6 (18.8)
Unknown	5 (11.1)	1 (3.1)

ECOG, Eastern Cooperative Oncology Group; PD-L1, programmed death-ligand 1.

The objective response rate (ORR) of the population included in this analysis was 65.6%, which was consistent with that of the whole CLAP population (online supplemental table 1). The median duration of the response, progression-free survival (PFS), and overall survival (OS) were not reached (online supplemental figure 1A, B).

10.1136/jitc-2020-002223.supp10 Supplementary data

### Specific genetic alterations and response to treatment

**Figure 1:**
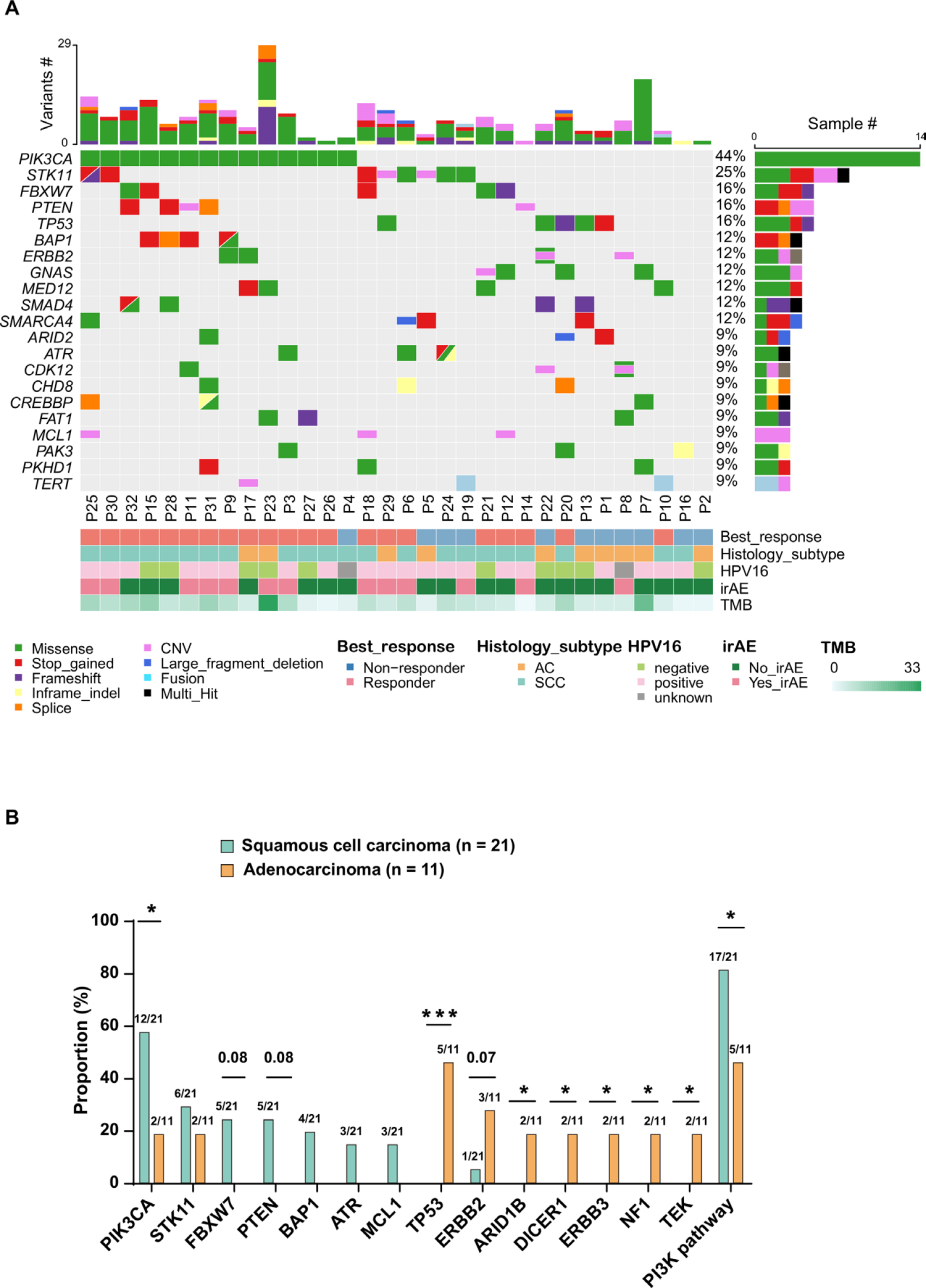
Distribution of genetic alterations with regard to clinical characteristics, response to treatment, and TMB (A). Association between genetic alterations and histological subtypes (B). CNV, copy number variation; irAE, immune-related adverse event; AC, adenocarcinoma; SCC, squamous cell carcinoma; TMB, tumor mutational burden; * < 0.05; *** < 0.001.

The most commonly altered genes (alteration frequency ≥9%) are presented in figure 1A, with their single-nucleotide substitution profile shown in online supplemental figure 2. Nearly half of the patients (43.8%) had *PIK3CA* missense mutations. Alterations in *STK11*, *FBXW7*, *PTEN*, and *TP53* were identified in 25%, 15.6%, 15.6%, and 15.6% of patients, respectively. Notably, genetic alterations in the PI3K/AKT pathway were found in 68.8% of the patients (online supplemental figure 3). Only one patient (3.1%) carried *CD274* (PD-L1) and *PDCD1LG2* (PD-L2) amplifications.

10.1136/jitc-2020-002223.supp2 Supplementary data

10.1136/jitc-2020-002223.supp3 Supplementary data

SCC and adenocarcinoma displayed distinct genetic alteration profiles (figure 1B). *PIK3CA* mutations (57.1% vs 18.2%, p=0.03) were significantly enriched in SCC, whereas alterations of *TP53*, *ARID1B*, *DICER1*, *ERBB3*, *NF1*, and *TEK* were exclusively detected in patients with adenocarcinoma in our cohort. Genetic alterations involved in the PI3K/AKT pathway were more common in patients with SCC than in adenocarcinoma (80.9% vs 45.5%, p=0.05). Lastly, there was a trend toward more patients with adenocarcinoma harboring *ERBB2* alterations (27.3% vs 4.8%, p=0.07).

The types of genetic alterations according to histological subtypes are presented in online supplemental figure 4A. Frameshift mutations were more commonly detected in patients with adenocarcinoma than SCC (18.2% vs 5.4%, p=0.001; online supplemental figure 4B).

10.1136/jitc-2020-002223.supp4 Supplementary data

**Figure 2:**
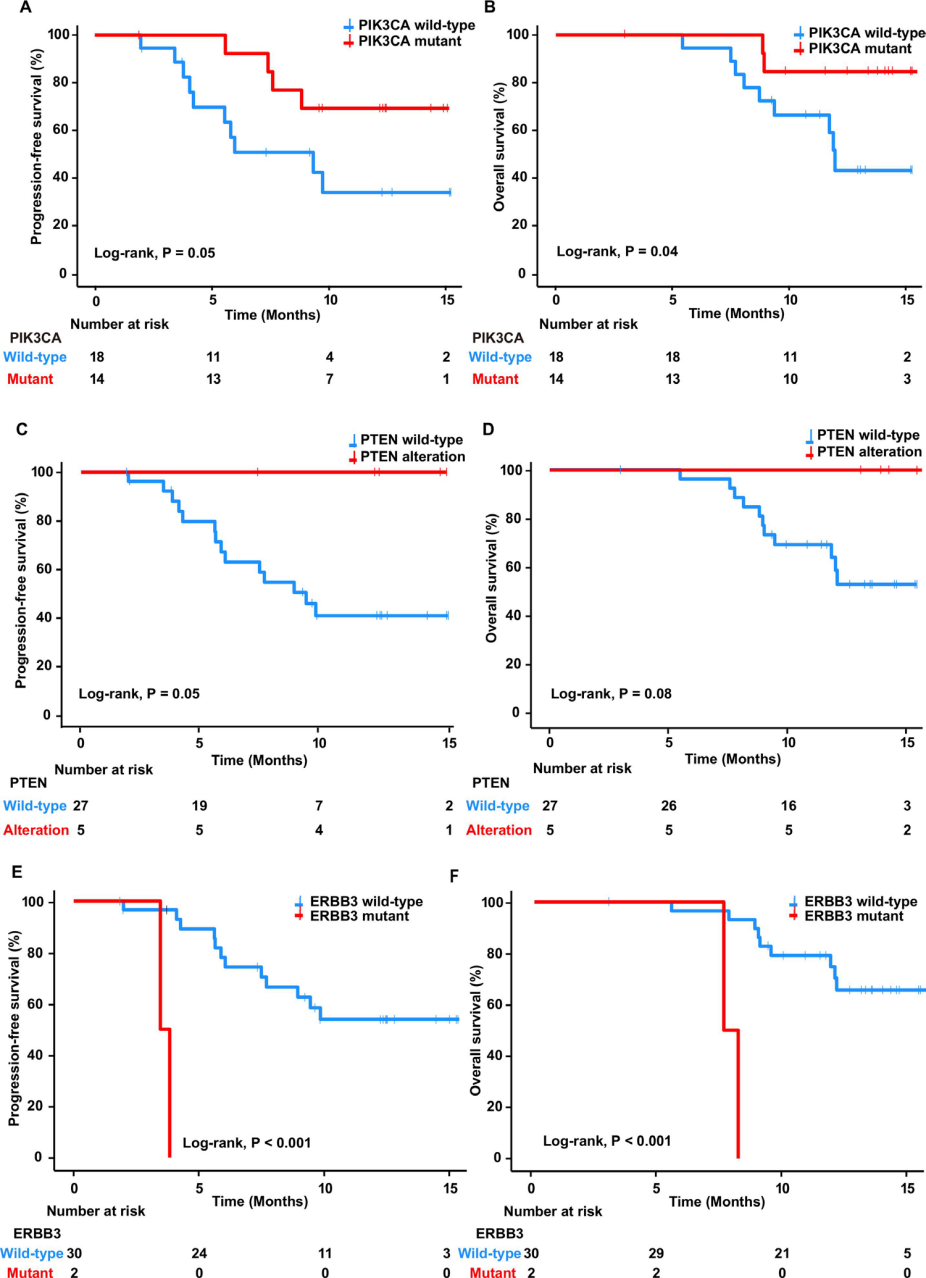
Kaplan-Meier curves of progression-free survival in patients with *PIK3CA* mutations and wild type (A), *PTEN* alteration and wild type (C), and *ERBB3* mutations and wild type (E). Kaplan-Meier curves of overall survival in patients with *PIK3CA* mutations and wild type (B), *PTEN* alteration and wild type (D), and ErbB3 mutations and wild type (F).

We assessed alterations in certain cancer-related genes to evaluate their influence on treatment response. Patients with *PIK3CA* mutations showed significantly longer median PFS and OS than those with wild-type *PIK3CA* (HR 0.33, 95% CI 0.10 to 1.05, p=0.05 and HR 0.23, 95% CI 0.05 to 1.08, p=0.04, respectively; figure 2A, B and table 2). Similarly, *PIK3CA* mutations were significantly higher in responders than in non-responders (61.9% vs 9.1%, OR 16.25, 95% CI 2.11 to 187.60, p=0.004; online supplemental figure 5A). Most *PIK3CA* mutations were found at E545K (50%) and E542K (28.6%). Moreover, *PTEN* alterations were associated with improved outcomes; it conferred longer PFS (HR 3.71^e-09^, 95% CI 0–Inf, p=0.05; figure 2C and table 2), with all patients achieving PFS of ≥6 months (online supplemental figure 5B). *PTEN* alterations trend toward increased OS (HR 3.64^e-09^, 95% CI 0–Inf, p=0.08; figure 2D and table 2) and higher ORR (23.8% vs 0%, OR Inf, 95% CI 0.76–Inf, p=0.08; online supplemental figure 5A) were also observed. Since alterations of *PIK3CA* and *PTEN* were associated with improved clinical outcome, we then investigated whether the genetic alterations in the PI3K/AKT pathway might improve the prediction of treatment response. Patients with gene alterations involved in the PI3K/AKT pathway had a significantly better median PFS (HR 0.33, 95% CI 0.11 to 0.94, p=0.03; table 2 and online supplemental figure 6A) and OS (HR 0.25, 95% CI 0.08 to 0.84, p=0.02; table 2 and online supplemental figure 6B), which suggested a superior predictive power of PI3K/AKT pathway genetic alterations to either *PIK3CA* and *PTEN* alterations alone. In contrast, two patients with *ERBB3* mutations had significantly shorter median PFS and OS than did *ERBB3* wild-type patients (HR 34.9, 95% CI 3.09 to 394.00, p˂ 0.001, and HR 19.8, 95% CI 2.71 to 144.00, p˂ 0.001, respectively; figure 2E, F and table 2); neither of them experienced responses (0% vs 18.2%, OR 0.00, 95% CI 0.00 to 1.08, p=0.04; online supplemental figure 5A). The two identified *ERBB3* mutations were at G284R and R1136C.

10.1136/jitc-2020-002223.supp5 Supplementary data

10.1136/jitc-2020-002223.supp6 Supplementary data

**Table:** Table 2. Univariate and multivariate analysis for progression-free survival and overall survival

Factors	Progression-free survival			Overall survival		
	Univariate analysis		Multivariate analysis	Univariate analysis		Multivariate analysis
	HR (95% CI)	*P* value	*P* value	HR (95% CI)	*P* value	*P* value
Histologic subtype						
Squamous cell carcinoma vs adenocarcinoma	0.28 (0.10–0.81)	0.01	0.25	0.33 (0.10–1.07)	0.05	0.54
*PIK3CA*						
Mutant vs Wild-type	0.33 (0.10–1.05)	0.05	0.15	0.23 (0.05–1.08)	0.04	0.78
*PTEN*						
Altered vs Wild-type	3.71^e-09^ (0–Inf)	0.05	0.41	3.64^e-09^ (0–Inf)	0.08	0.77
*ERBB3*						
Mutant vs Wild-type	34.9 (3.09–394.00)	˂ 0.001	0.01	19.8 (2.71–144.00)	˂ 0.001	0.02
PI3K/AKT pathway						
Altered vs Wild-type	0.33 (0.11–0.94)	0.03	0.09	0.25 (0.08–0.84)	0.02	0.97
PD-L1 expression						
Positive vs negative	0.36 (0.11–1.19)	0.08	0.01	0.31 (0.09–1.06)	0.05	0.02
TMB						
≥5 mut/Mb vs<5 mut/Mb	0.26 (0.09–0.77)	0.01	0.01	0.31 (0.09–1.07)	0.05	0.18

CI, confidence interval; HR, hazard ratio; mut/Mb, mutations per megabase; PD-L1, programmed death-ligand 1; TMB, tumor mutational burden.

Finally, we used The Cancer Genome Atlas (TCGA) public database to investigate the potential roles of the gene signatures mentioned previously in cervical cancer. In total, data from 267 patients with cervical cancer were available, of which 202 patients were from the study by the Cancer Genome Atlas Research Network.^15^ Patient characteristics of the TCGA data set are shown in online supplemental table 2. In the TCGA data set, the median age was 47 years, with 78.3% of the patients aged ˂ 60 years. Similar to our cohort, most patients (89.5%) in the TCGA data set had SCC. More than half of the patients were diagnosed with stage I cancer. None of the aforementioned genetic markers had an impact on the OS (online supplemental figure 7).

10.1136/jitc-2020-002223.supp11 Supplementary data

10.1136/jitc-2020-002223.supp7 Supplementary data

### TMB associated with response to treatment

**Figure 3:**
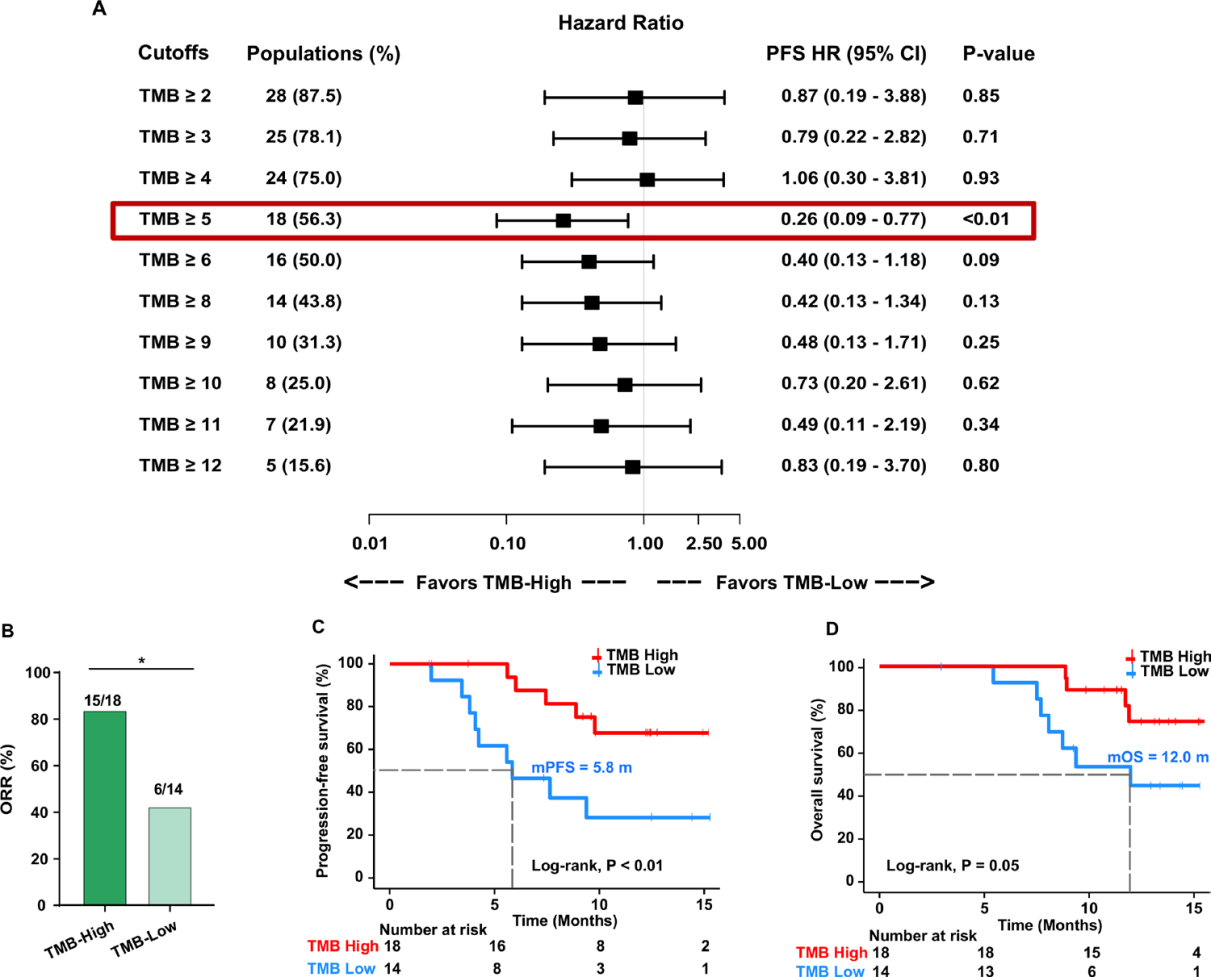
Cut-off selection of TMB associated with PFS (A). Association between TMB levels and ORR (B). Kaplan-Meier curves of PFS (C) and overall survival (D) in patients with high TMB and low TMB. ORR, objective response rate; PFS, progression-free survival; TMB, tumor mutational burden. *< 0.05.

We used different cutoffs to identify TMB value thresholds that defined TMB-high vs TMB-low groups and investigated the association between TMB levels and treatment response. We found that the cut-off value of 5 mutations per megabase (mut/Mb) of TMB could effectively separate patients with high and low TMB (figure 3A). The RR was significantly higher in patients with TMB-high (≥5 mut/Mb) than in those with TMB-low (83.3% vs 42.9%, OR 6.67, 95% CI 1.45 to 27.65, p=0.03; figure 3B). Moreover, the median PFS and OS were significantly longer in patients with TMB-high than in those with TMB-low (5.83 vs not reached (NR) months, HR 0.26, 95% CI 0.09 to 0.77, p<0.01, and 11.97 vs NR months, HR 0.31, 95% CI 0.09 to 1.07, p=0.05, respectively; figure 3C, D and table 2).

### TMB combined with PD-L1 expression

In the CLAP trial, positive PD-L1 expression correlated with prolonged PFS and OS. Consistent with the CLAP trial, patients with positive PD-L1 expression in the current cohort showed significantly better survival (PFS, HR 0.36, 95% CI 0.11 to 1.19, p=0.08; OS, HR 0.31, 95% CI 0.09 to 1.06, p=0.05; table 2; online supplemental figure 8A and 8B). We then explored whether TMB combined with PD-L1 expression could further predict clinical benefit. We divided patients into four subgroups based on the combination of TMB status and PD-L1 expression (TMB-high/PD-L1-positive, TMB-low/PD-L1-positive, TMB-high/PD-L1-negative, and TMB-low/PD-L1-negative). The ORR was 81.3% in the TMB-high/PD-L1-positive subgroup, while it was only 25% in the TMB-low/PD-L1-negative subgroup (online supplemental figure 8C). The median PFS and OS in TMB-high/PD-L1-positive subgroup were remarkably longer than those in the TMB-low/PD-L1-negative subgroup (PFS, NR vs 4.9 months, HR 0.12, 95% CI 0.008 to 1.78, p<0.001; OS, NR vs 8.2 months, HR 0.13, 95% CI 0.01 to 1.60, p=0.003; log-rank test for the two subgroups; online supplemental figure 8D and 8E), suggesting that the combination of TMB status and PD-L1 expression has a better prediction value.

10.1136/jitc-2020-002223.supp8 Supplementary data

### Multivariate COX regression analysis

Univariate analysis showed improved PFS and OS in patients with SCC compared with adenocarcinoma (HR 0.28, 95% CI 0.10 to 0.81, p=0.01, and HR 0.33, 95% CI 0.10 to 1.07, p=0.05, respectively; table 2). Therefore, histological subtype was included in the multivariate analysis with all the aforementioned molecular features (table 2). Of these, *ERBB3* mutations were significantly associated with poor PFS and OS (p=0.01 and p=0.02, respectively). Patients with positive PD-L1 expression showed a significantly longer PFS and OS (p=0.01 and p=0.02, respectively). Similarly, TMB-high also correlated with superior survival (PFS, p=0.01, and OS, p=0.18, respectively).

### Gene alterations and immune-related adverse events (irAEs)

**Figure 4:**
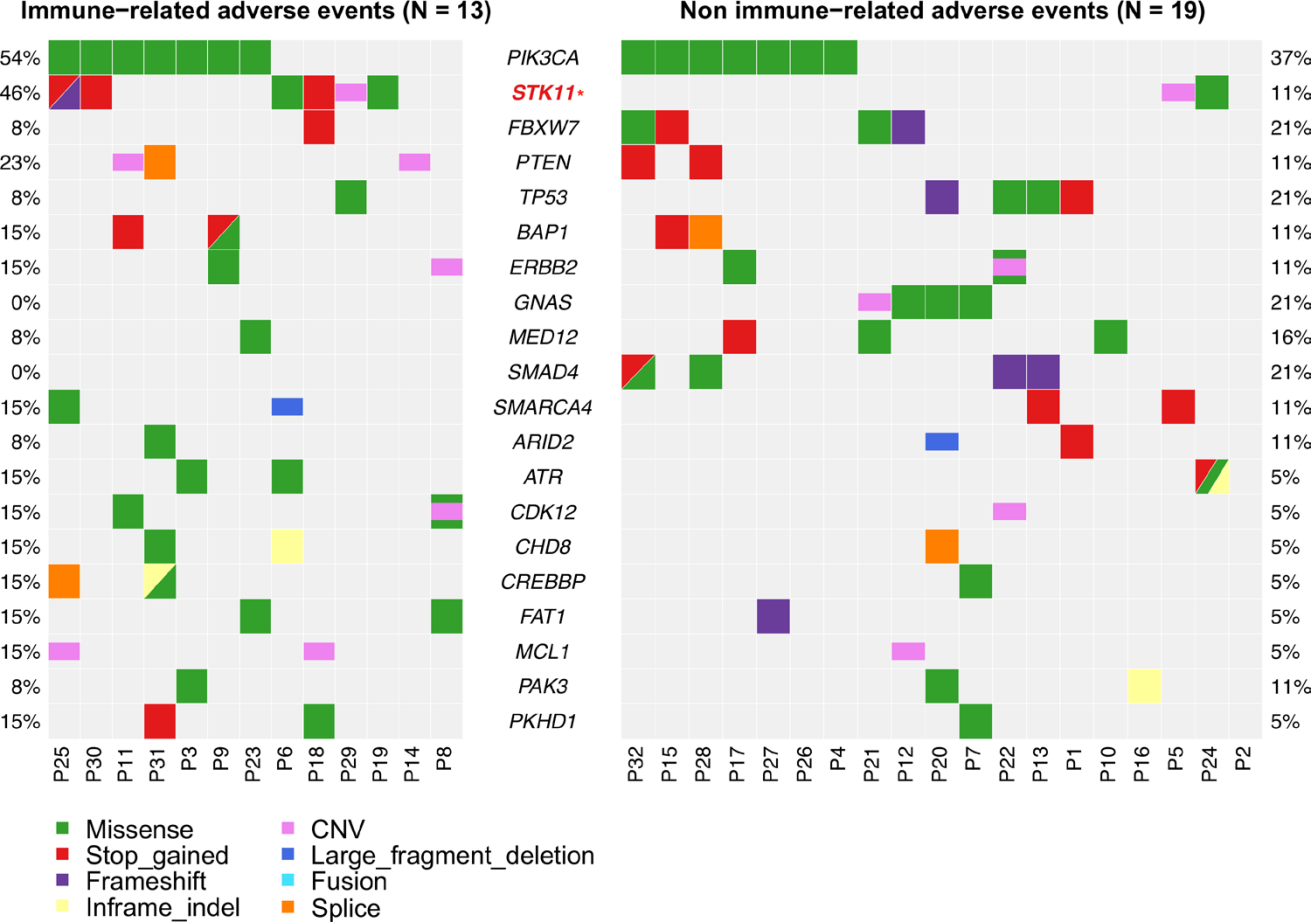
Distribution of genetic alterations with regard to the occurrence of immune-related adverse events. CNV, copy number variation.

Interestingly, we found that *STK11* alterations were significantly higher in patients with irAEs than in those without (46.2% vs 10.5%, p=0.02; figure 4). In addition, *GNAS* and *SMAD4* alterations were enriched in patients without irAEs (21.1% vs 0%, p=0.08, and 21.1% vs 0%, p=0.08, respectively; figure 4), with no *GNAS* and *SMAD4* alterations in patients with irAEs.

## Discussion

In this study, we investigated the predictive value of genomic profiling and TMB in Chinese patients with cervical cancer treated with PD-1 and VEGFR-2 inhibitors in the phase II CLAP trial. The PI3K/AKT pathway and *ERBB3* genetic alterations were associated with clinical outcomes in our ICI-treated cohort. Given that there is no difference in survival in the TCGA cohort between these gene mutations and the wild type, where patients did not receive immunotherapy, our findings support the role of these biomarkers in predicting the response to combination immunotherapy. To the best of our knowledge, this is the first study on genomic profiling of advanced cervical cancer to identify potential predictors of response to ICI combination therapy.

In this study, alterations in the PI3K/AKT pathway genes, particularly *PIK3CA* and *PTEN*, were associated with favorable clinical outcome. Although genetic alterations in the PI3K/AKT pathway have been reported to be associated with immunotherapy resistance,^16 17^ emerging evidence revealed that the activation of the PI3K/AKT pathway may be a good target for immunotherapy. Parsa *et al* demonstrated that the expression of PD-L1 increased post-transcriptionally after loss of PTEN and activation of the PI3K/AKT pathway in glioma.^18^ In addition, recurrent neoantigens were identified in *PIK3CA* mutations across several cancer types.^19^ Recently, Nusrat *et al* reported that patients with microsatellite stable colorectal cancer with *PIK3CA* mutations in seven immunotherapy trials had favorable clinical outcomes from immunotherapy.^20^ They found that half of patients with *PIK3CA* mutations derived clinical benefit compared with 8.6% *PIK3CA* wild-type patients (p=0.015), with a trend toward longer time to progression in *PIK3CA* mutation patients (3.8 months in mutation patients vs 2.1 months in wild type, p=0.08). Consistent with these data, we observed that patients with advanced cervical cancer bearing gene alterations in the PI3K/AKT pathway were associated with a better response to PD-1 inhibitor combination therapy.

ERBB3, also known as HER3, is a member of the ErbB family of receptor tyrosine kinases and a homolog of EGFR and HER2. Previous data suggested that ICI monotherapy showed limited benefit, but the combination of ICI therapy with chemotherapy had a promising effect in patients with EGFR-mutant non-small-cell lung cancer (NSCLC).^21 22^ In the current study, patients with *ERBB3* mutations had a significantly decreased response to ICI combination therapy, with a trend toward poor PFS and OS in patients with genetic alterations in the ErbB family (online supplemental figure 9). It has been shown that *ERBB3* activation requires the formation of heterodimers with other ErbB family receptors (mainly HER2 and EGFR) owing to its impaired protein kinase activity. Additionally, the role of *ERBB3* mutations in immune escape was reported to be mediated through the PI3K/AKT/mTOR pathway.^23^ However, only one of the two patients with *ERBB3* mutations in our cohort carried *ERBB2* alterations simultaneously, and none harbored genetic alterations in the PI3K/AKT pathway. We had no plausible explanation for these findings; thus, the molecular mechanism should be further explored.

10.1136/jitc-2020-002223.supp9 Supplementary data

TMB has been demonstrated as a predictor for ICI response in numerous studies.^24 25^ Fang *et al* confirmed the accuracy and the predictive value of TMB as assessed by the targeted gene panel employed in the current study.^26^ However, determination of the cutoffs for TMB in various cancer types is a significant issue in clinical assessment. For targeted panel-based TMB in NSCLC, the cutoffs were set at approximately 10 mut/Mb for FoundationOne CDx panel and 7.4 mut/Mb for Memorial Sloan Kettering-Integrated Mutation Profiling of Actionable Cancer Targets panel, with 9.65 mut/Mb (median) and 16 mut/Mb (upper quartile), respectively, in urothelial carcinoma.^27–30^ In the current study, we found that the threshold for TMB at 5 mut/Mb identified patients with distinct responses to treatment. To our knowledge, this is the first TMB estimation study in patients with advanced cervical cancer treated with PD-1 combination therapy. Furthermore, as TMB and PD-L1 are two independent predictors of immunotherapy, we found that patients in the TMB-low/PD-L1-negative subgroup had impaired outcomes compared with TMB-low patients. These findings suggest that the combined TMB and PD-L1 expression provides further information on predicting response to PD-1 inhibitor combination therapy, particularly in patients who are likely to be non-responders.

Interestingly, we found *STK11* alterations were enriched among patients occurring irAEs. Despite *STK-11* alterations linked to a lack of response to ICI therapy,^31^ the association between *STK-11* alterations and the occurrence of irAEs had not been reported. However, the small number of patients precluded a definite conclusion.

Our study has several limitations. First, our findings were not validated in an independent cohort; validation of our results in an independent cohort is warranted to assess the robustness of our results. Nevertheless, an independent cohort is not yet available owing to the lack of public genomic profiling data in cervical cancer ICI studies. Second, even though our results supported the predictive value of these identified genetic markers, we cannot exclude the possibility that they are prognostic factors. All patients in our cohort had advanced cervical cancer and were selected as appropriate candidates in our trial. The differences in baseline characteristics between the two cohorts may yield selection bias. We acknowledge that the predictive role of our findings await confirmation by future clinical trials.

## Conclusions

In summary, we uncovered that genetic alterations in *PIK3CA*, *PTEN*, *ERBB3*, and PI3K/AKT pathway could be novel predictive biomarkers in patients with cervical cancer treated with PD-1 inhibitor combination therapy. Furthermore, we demonstrated the predictive value and assigned a threshold for TMB assessed using the gene panel in advanced cervical cancer.

## Data Availability

Data are available upon reasonable request. Data may be obtained from a third party and are not publicly available. The key raw data have been recorded at Research Data Deposit public platform (http://www.researchdata.org.cn) with number RDDB2020001016. The data are available from Research Data Deposit, but restrictions apply to the availability of these data, which were used under license for the current study and so are not publicly available. However, data are available from the authors upon reasonable request and with permission of the Research Data Deposit public platform.
