# Correction: *Genomic profiling of advanced cervical cancer to predict response to programmed death-1 inhibitor combination therapy: a secondary analysis of the CLAP trial*

**DOI:** 10.1136/jitc-2020-002223corr1

**Published:** 2022-02-16

Huang X, He M, Peng H, *et al*. Genomic profiling of advanced cervical cancer to predict response to programmed death-1 inhibitor combination therapy: a secondary analysis of the CLAP trial. *J Immunother Cancer* 2021;**9:**e002223.

This article has been corrected since it was first published. It now states that authors Xin Huang, Minjun He, Hongyu Peng, Chongjie Tong and Zhimin Liu contributed equally.

