# Supplementary figures and images for: Genomic profiling of advanced cervical cancer to predict response to programmed death-1 inhibitor combination therapy: a secondary analysis of the CLAP trial
Source: J Immunother Cancer. 2021 May 17;9(5):e002223. doi: 10.1136/jitc-2020-002223 (PMC8137235; doi:10.1136/jitc-2020-002223)

Suppl. Fig 3

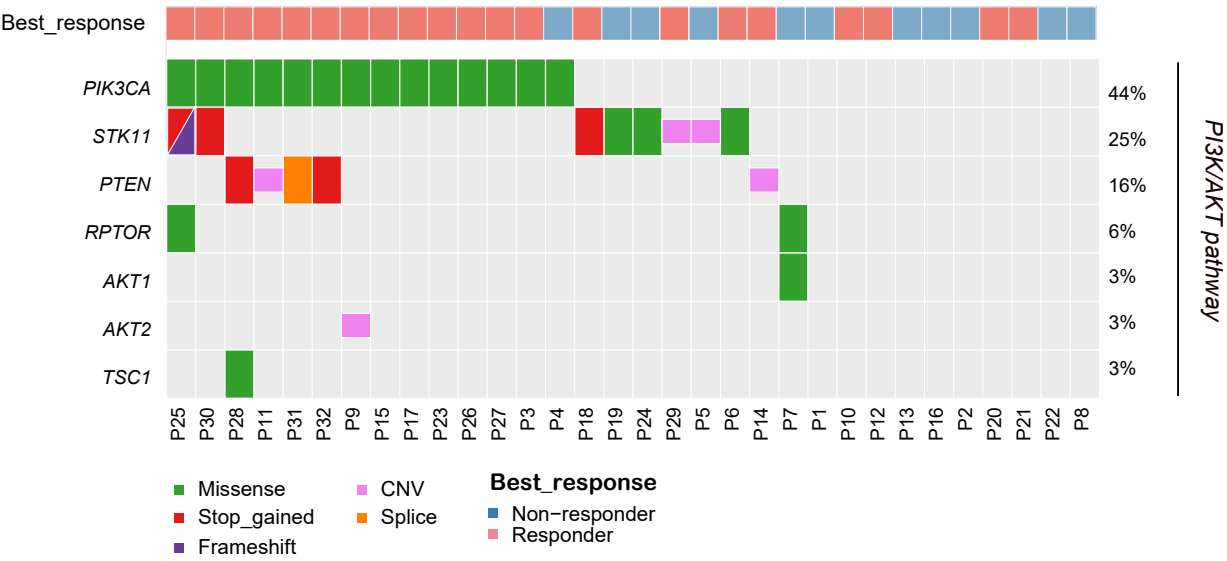

Supplement: Supplementary data [file jitc-2020-002223supp001.pdf]

Suppl. Fig.1

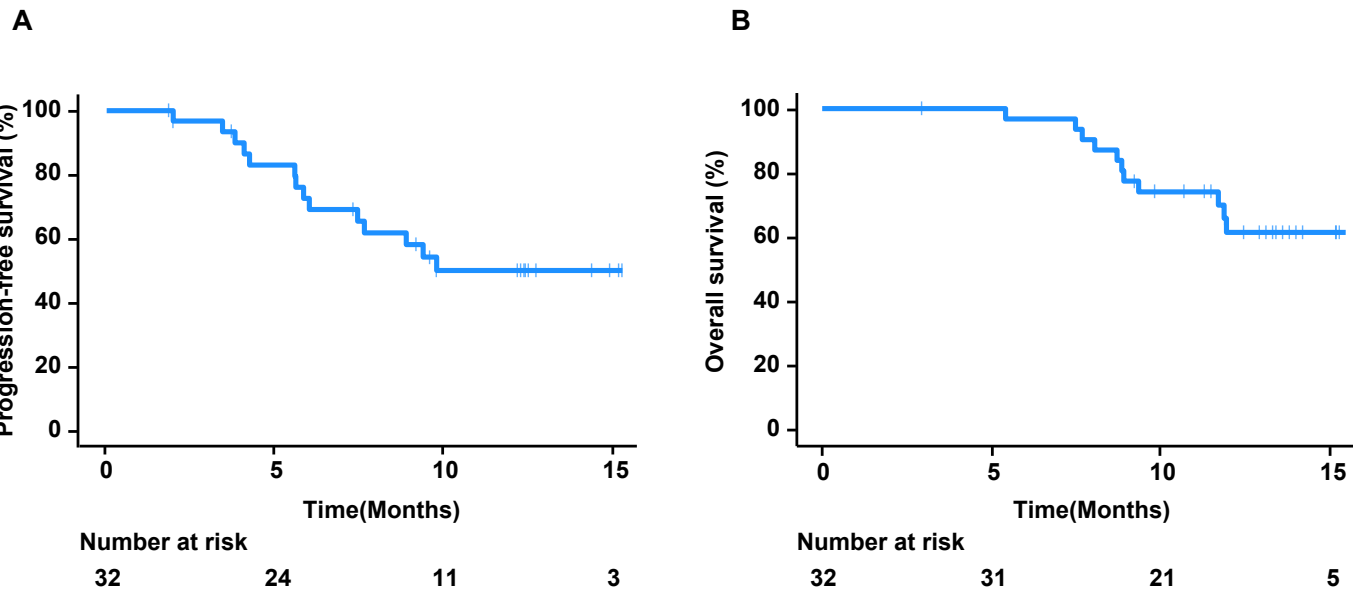

Supplement: Supplementary data [file jitc-2020-002223supp002.pdf]

Suppl. Fig 2

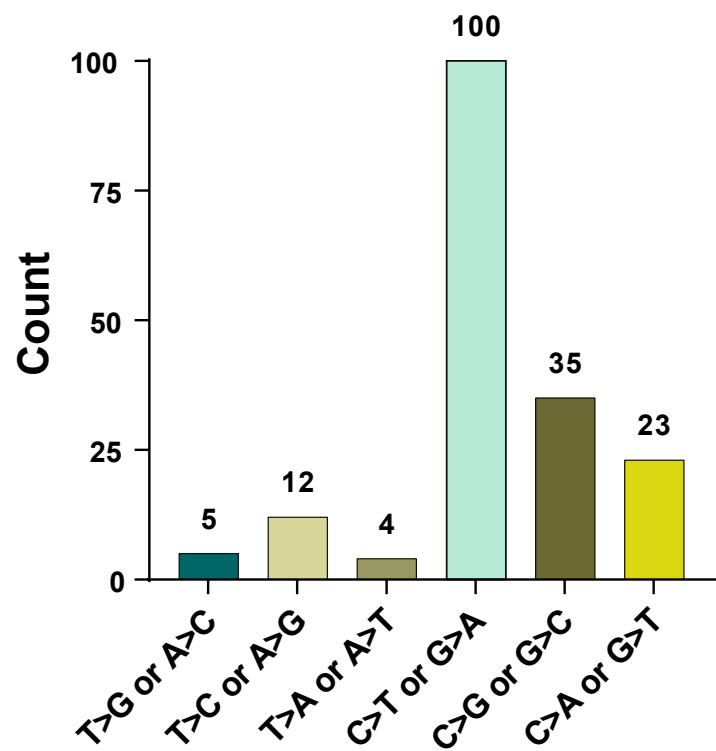

Supplement: Supplementary data [file jitc-2020-002223supp003.pdf]

Suppl. Fig 4

A

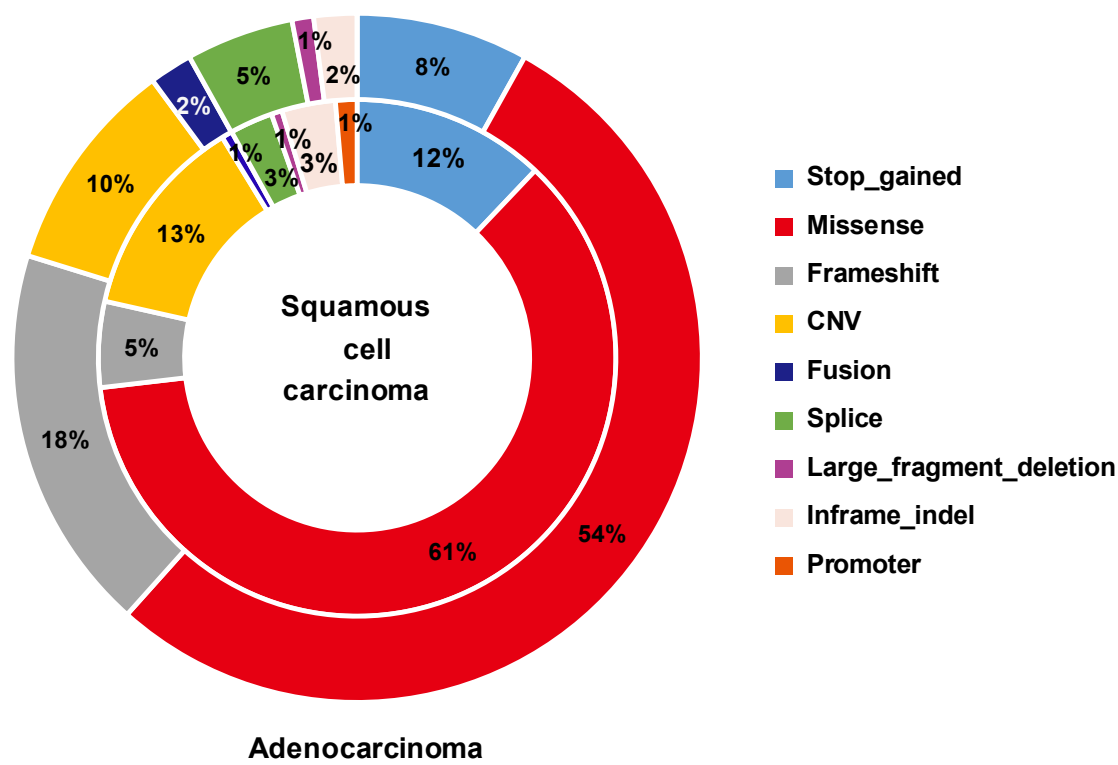

B

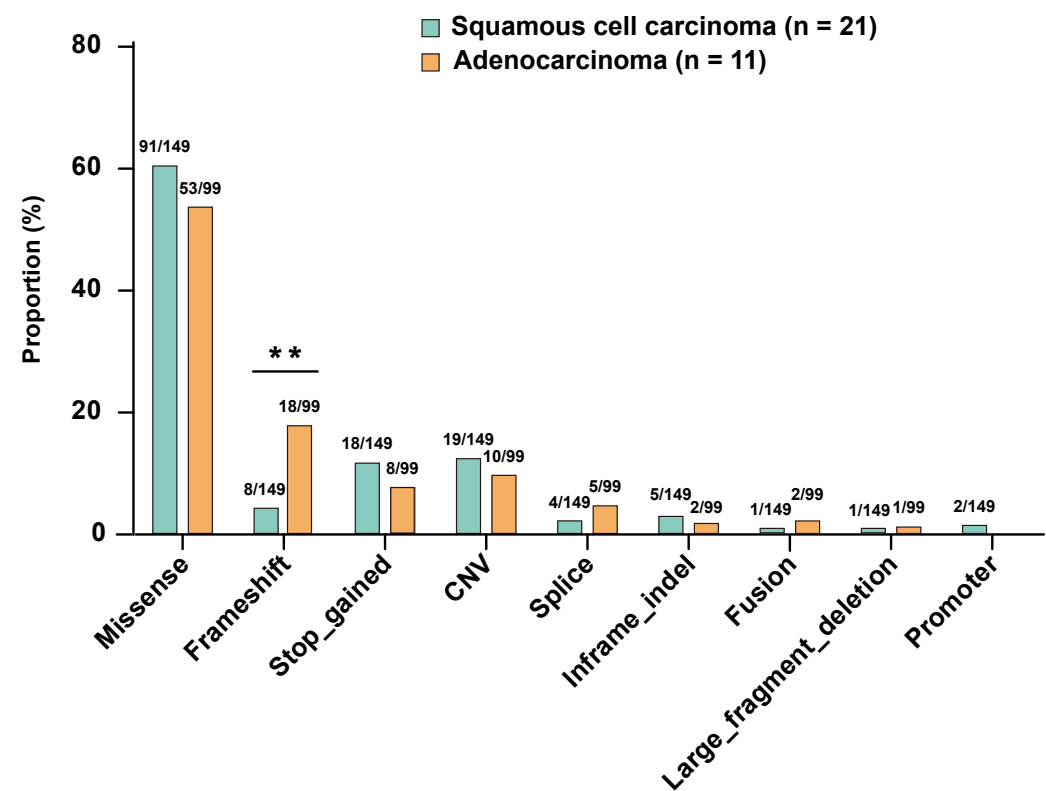

Supplement: Supplementary data [file jitc-2020-002223supp004.pdf]

Suppl. Fig 5

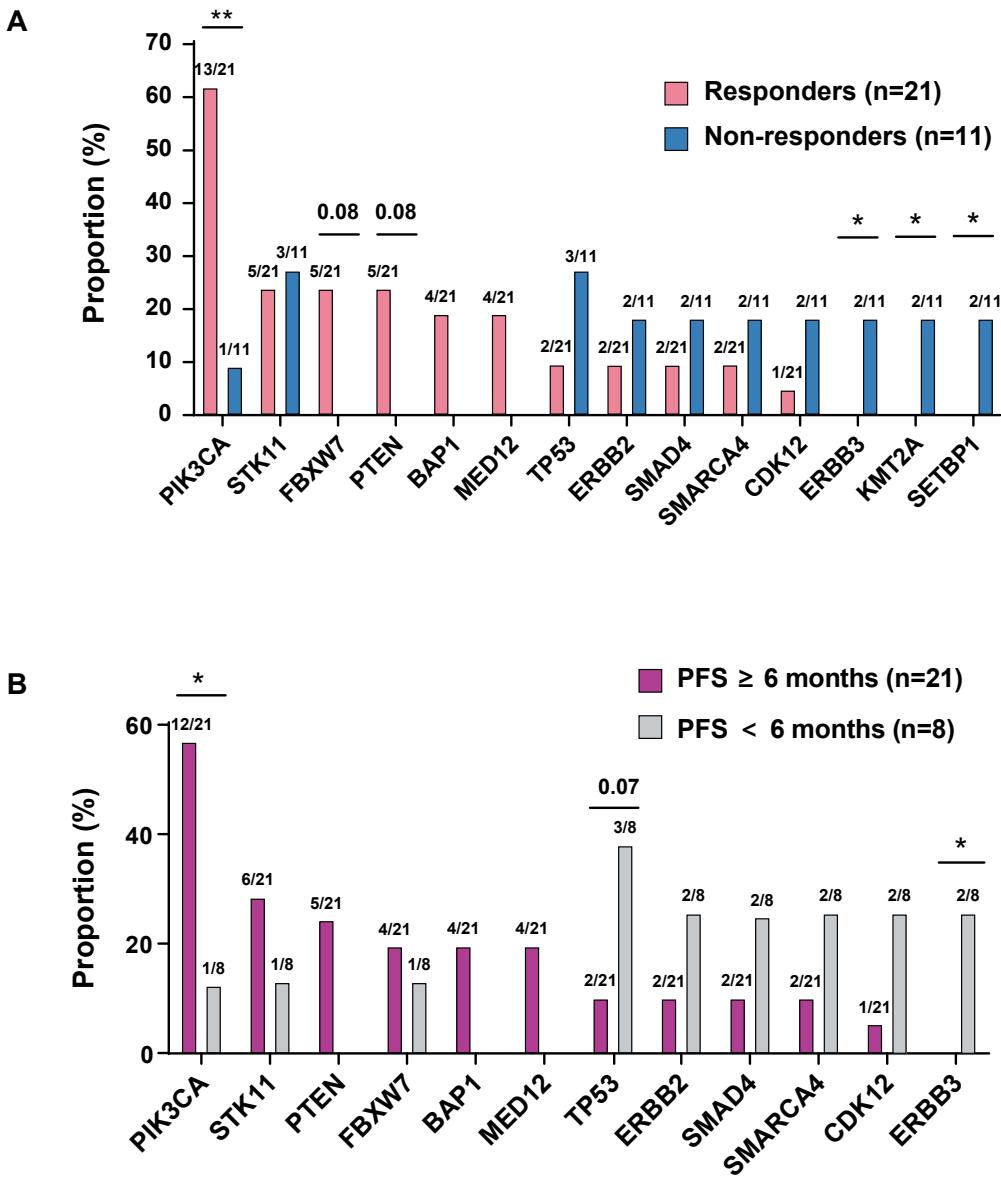

Supplement: Supplementary data [file jitc-2020-002223supp005.pdf]

Suppl. Fig 6

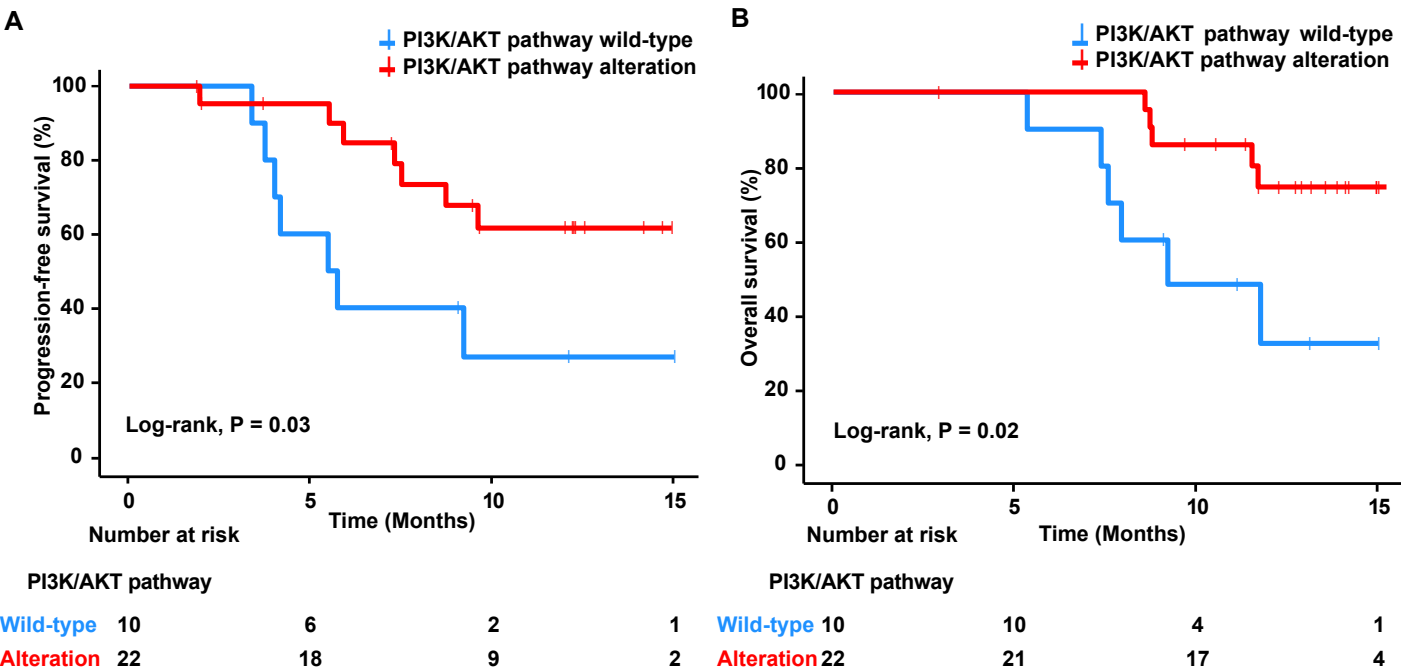

Supplement: Supplementary data [file jitc-2020-002223supp006.pdf]

Suppl. Fig 7

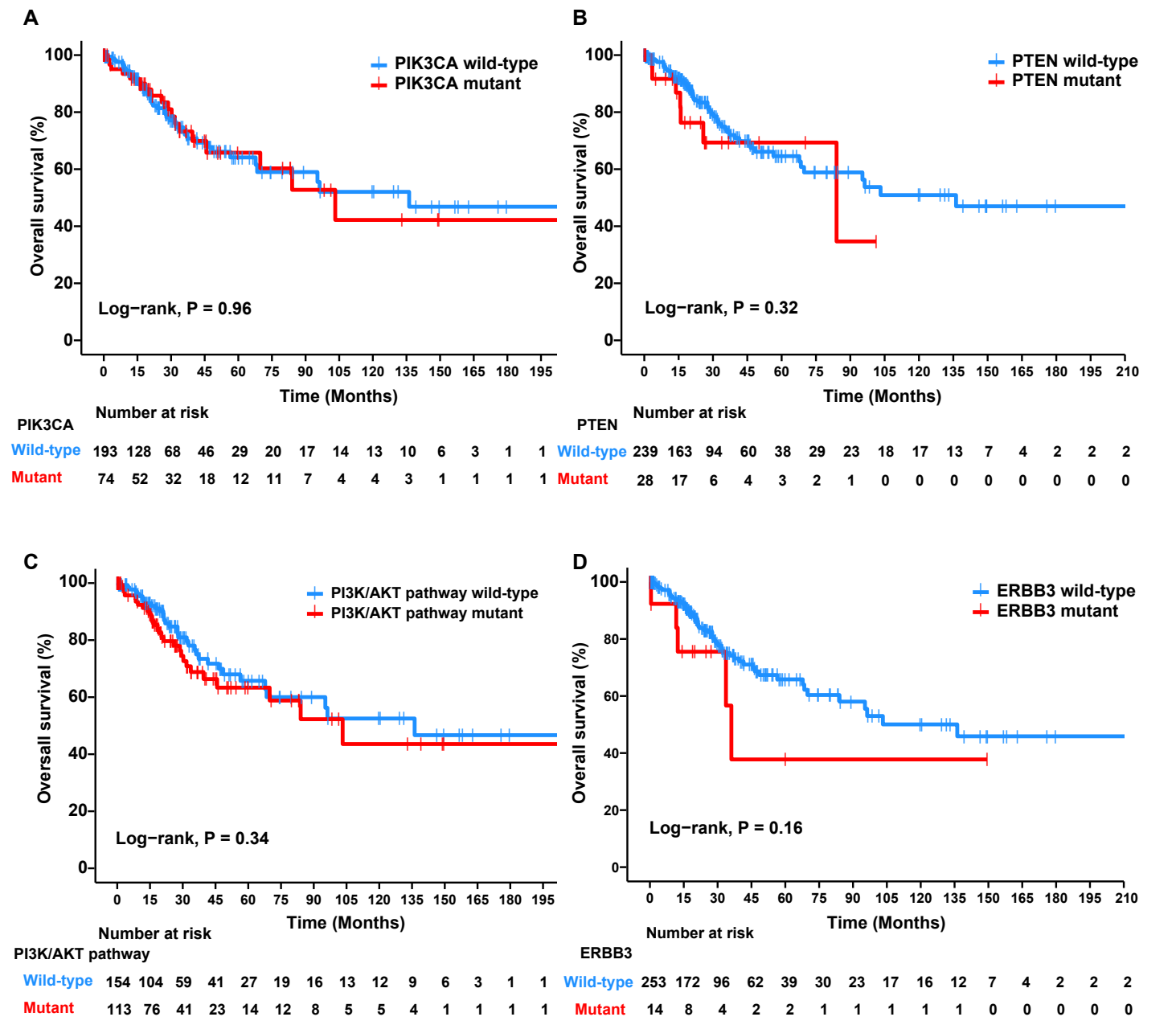

Supplement: Supplementary data [file jitc-2020-002223supp007.pdf]

Suppl. Fig 8

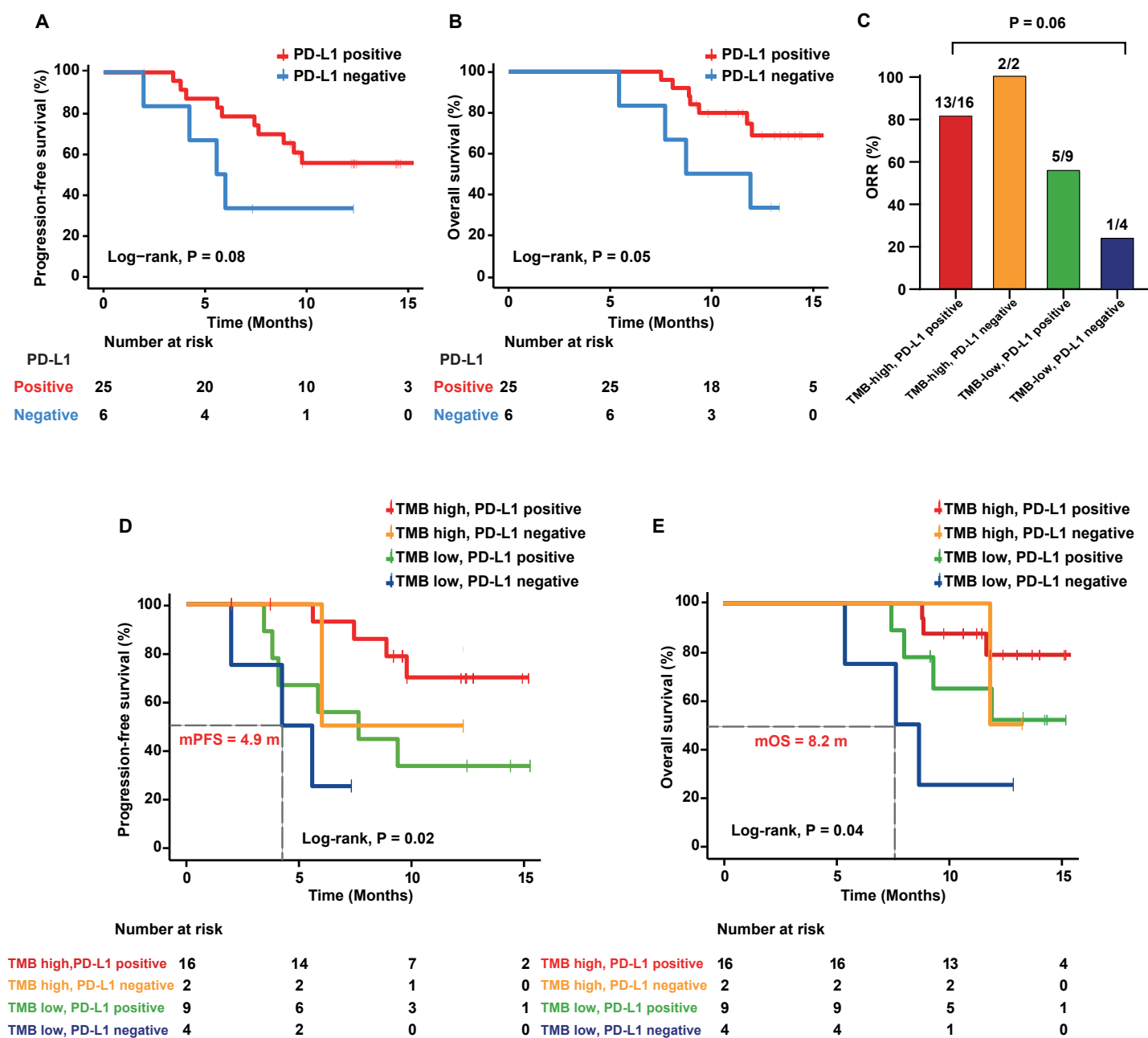

Supplement: Supplementary data [file jitc-2020-002223supp008.pdf]

Suppl. Fig 9

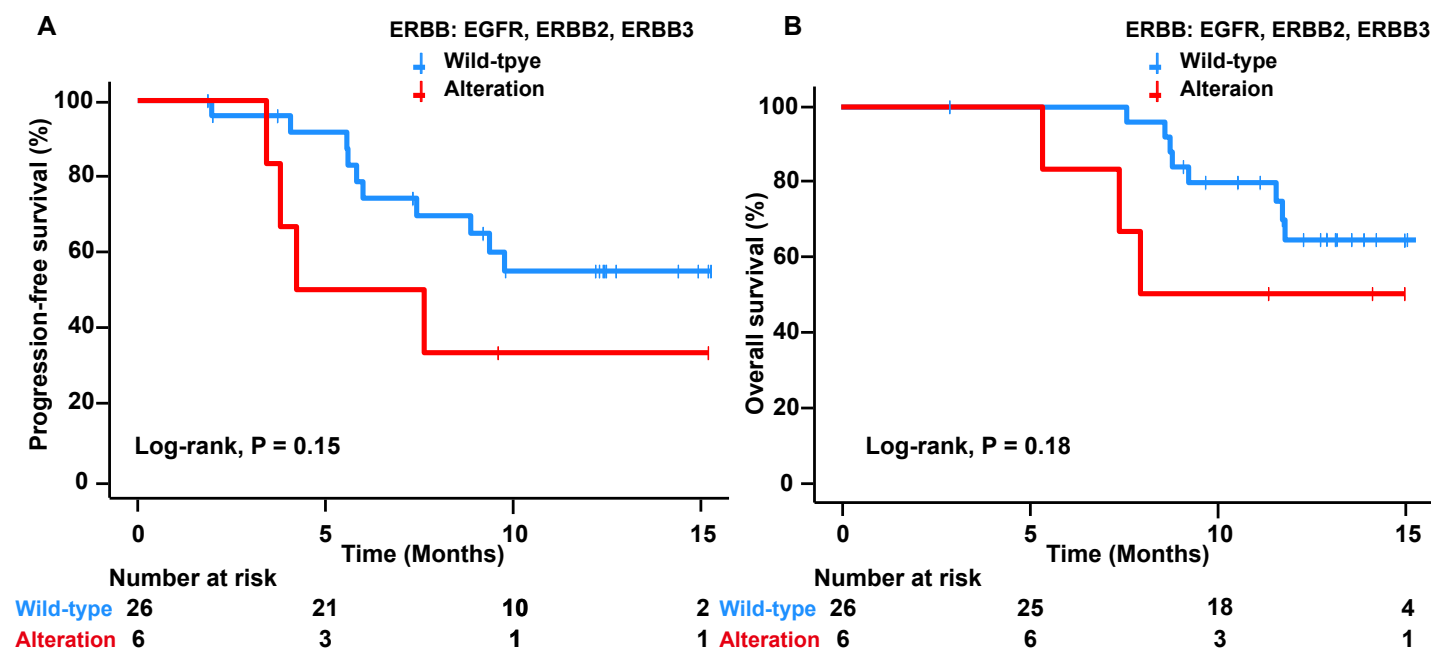

Supplement: Supplementary data [file jitc-2020-002223supp009.pdf]
