# Supplementary material for: Genomic profiling of advanced cervical cancer to predict response to programmed death-1 inhibitor combination therapy: a secondary analysis of the CLAP trial
Source: J Immunother Cancer. 2021 May 17;9(5):e002223. doi: 10.1136/jitc-2020-002223 (PMC8137235; doi:10.1136/jitc-2020-002223)
Supplement: Supplementary data [file jitc-2020-002223supp010.pdf]

Suppl. Table 1 Antitumor activity in the CLAP study and in the current analysis

Antitumor Activity	CLAP study population (*N* = 45)	Subpopulation in the current analysis (*N* = 32)
ORR	25 (55.6)	21 (65.6)
DCR	37 (82.2)	30 (93.8)
Best overall response		
CR	2 (4.4)	2 (6.3)
PR	23 (51.1)	19 (59.4)
SD	12 (26.7)	9 (28.1)
Progressive disease	4 (8.9)	2 (6.3)
Not available	4 (8.9)	–

Data are presented as No. (%), unless otherwise specified.

ORR, objective response rate; DCR, disease control rate; CR, complete response; PR, partial response; SD, stable disease.
