# Supplementary material for: Genomic profiling of advanced cervical cancer to predict response to programmed death-1 inhibitor combination therapy: a secondary analysis of the CLAP trial
Source: J Immunother Cancer. 2021 May 17;9(5):e002223. doi: 10.1136/jitc-2020-002223 (PMC8137235; doi:10.1136/jitc-2020-002223)
Supplement: Supplementary data [file jitc-2020-002223supp011.pdf]

Suppl. Table 2 Patient characteristics available from TCGA database

Characteristics	TCGA population *N* (%)
Patients	*N*=267
Age (years)	
< 60	209 (78.3)
≥ 60	56 (21.0)
Unknown	2 (0.8)
Median (range)	47 (21–90)
Histology	
Squamous cell carcinoma	239 (89.5)
Adenocarcinoma	28 (10.5)
FIGO stage	
Stage I	142 (53.2)
Stage II	60 (22.5)
Stage III	42 (15.7)
Stage IV	16 (6.0)
Unknown	7 (2.6)
